# Normal Dual Isotope V/Q SPECT Model for Monte-Carlo Studies

**DOI:** 10.3389/fmed.2020.00461

**Published:** 2020-08-18

**Authors:** David Bourhis, Laura Wagner, Marine Essayan, Philippe Robin, Romain Le Pennec, Pierre Yves Salaun, Pierre Yves Le Roux

**Affiliations:** ^1^Service de Médecine Nucléaire, Centre Hospitalier Régional Universitaire de Brest, Brest, France; ^2^EA3878 GETBO, Université de Bretagne Occidentale, Brest, France

**Keywords:** V/Q SPECT, lung function, PE, simulation, Z-score

## Abstract

**Background:** There is currently no reliable or validated tool to delineate and quantify functional lung volumes with ventilation/perfusion (V/Q) SPECT/CT. The main challenges encountered include the physiological non-uniformity of lung function, such as the anterior-to-posterior gradient on perfusion images, and the lack of ground truth to assess the accuracy of delineation algorithms. In that respect, Monte-Carlo simulations would be an interesting tool. Thus, the aim of this study was to develop a realistic model of dual-isotope lung V/Q SPECT-CT Monte-Carlo simulations, integrating the anterior to posterior gradient on perfusion.

**Methods:** Acquisitions and simulations parameters were set in accordance to nuclear medicine guidelines for V/Q lung SPECT-CT. Projections were acquired and simulated, then the reconstructions [with and without attenuation correction (AC)] were compared. A model was built from a patient's CT scan. To model the anterior to posterior gradient, the lungs were divided into sixteen coronal planes, where a rising radioactivity concentration was set. To assess the realism of simulations, they were compared to a normal co-registered normal cases database in terms of pixelwize Z-score map.

**Results:** For ventilation images, mean (SD) Zscores on Zscore maps were −0.2 (0.7) and −0.2 (0.7) for AC and noAC images, respectively. For perfusion images, mean (SD) Zscores were −0.2 (0.6) and −0.1 (0.6) for AC and noAC images, respectively.

**Conclusion:** We developed a model for dual isotopes lung V/Q SPECT-CT, integrating the anterior-to-posterior gradient on perfusion images. This model could be used to build a catalog of clinical scenarios, in order to test delineation methods of functional lung volumes.

## Introduction

Lung function evaluation mainly relies on pulmonary function tests, which provide information about global lung function. Lung ventilation/perfusion scintigraphy is an imaging modality that provides complementary information about the regional distribution of lung function. Ventilation is assessed using inert gases or radio-labeled aerosols while local pulmonary blood flow is assessed after administration of ^99m^Tc labeled albumin macro-aggregates (MAA) (1, 2). Quantification of lung functional volumes demonstrated a potential utility in various clinical scenarios. This includes the quantification of the pulmonary vascular obstruction index (PVOI) in patients with pulmonary embolism (PE), which has been shown to be predictive of PE reccurrence, either at diagnosis or after anticoagulant therapy (3–5). Another indication is radiation oncology (6). The last 20 years have seen the emergence of new methods of irradiation (IMRT, VMAT, stereostatic radiotherapy), increasing the possibility of individualizing radiotherapy treatment plans, in order to increase the intensity to the tumor and/or to preserve lung function and limit the risk of pneumonitis (7, 8).

Although lung scintigraphy is a very attractive tool for regional lung function assessment, the main factor limiting a wider use of the test in clinical practice is the lack of reliable and validated tools to delineate and quantify functionnal lung volumes. Functionnal lung delineation is not trivial because of the physiological non-uniformity of lung ventilation and blood flow, mainly because of anatomy and gravity (9), leading to an anterior-to-posterior intensity gradient on perfusion SPECT. This makes delineation methods based on pixel intensities inaccurate, as an intensity value could be normal in the ventral area or pathological in the dorsal area, even if a quantified reconstruction is used. Several original segmentation methods have been proposed (10–13), but studies are often limited by the lack of ground truth.

Monte-Carlo simulations would be an intersting tool for assessing and validating a delineation algorithm for lung functional volumes with V/Q SPECT/CT. First, the advantage of simulating realistic lung V/Q SPECT-CT would be to know the ground truth for the evaluation and comparison of delineation methods (e.g., the exact size of a perfusion defect for PE, the exact pulmonary vascular obstruction index). Second, it would allow to test a much greater number of clinical scerarios (e.g., PE of various size and location). Finally, simulations may integrate physiogical parameters, such as gravity, which make conventional delineation methods inacurate in some situations.

Monte-Carlo simulations have been widely proposed in SPECT for multiple objectives (14). However, only a few studies have been published in the area of lung V/Q SPECT (12, 13, 15–17), and modeled the lung segmentation (18–22), but none have modeled the the anterior to posterior gradient on perfusion images. Furthermore, no study has modeled a dual energy ^81m^Kr gas ventilation associated with ^99m^Tc-MAA perfusion SPECT.

The aim of this study was to develop a realistic model of dual-isotopes lung V/Q SPECT-CT.

## Materials and Methods

### Data Acquisition and Simulation Parameters

All acquisitions were performed on a Symbia T6 system (Siemens, Erlangen) equipped with a medium energy low penetration (MELP) collimator, as it is used for dual-isotopes lung VQ SPECT-CT (1). SPECT were acquired with 4.7 mm pixels, 128 projections, 10 s/projections, step&shoot mode, non-circular orbit, in accordance with nuclear medicine clinical guidelines (1, 23). Energy windows for ^99m^Tc scatter, ^99m^Tc photopeak, ^81m^Kr scatter, and ^81m^Kr photopeak were [109.9, 129.5], [129.5, 150.5], [150.7, 177.6], [177.6, 206.4], respectively. CT acquisitions parameters were 110kV, 16 mAs with automatic exposure, pitch1. Attenuation correction CT (ACCT) reconstruction parameters were 512^2^ matrix, 0.98 mm pixel, 5 mm slice width and B08 filter. SPECT reconstructions were performed on Siemens MI-Apps software with FLASH3D (OSEM3D with collimator detector response modeling), 4 iterations, 8 subsets, 8.4 mm gaussian post-filtering, scatter correction [dual energy window method (DEW)], with and without attenuation correction.

The Monte-Carlo package used to run SPECT simulations was Simind (6.0) (24). The camera modeling parameters were set in order to correspond to a Symbia T6 system (Siemens, Erlangen) (25) equipped with a MELP collimator. All mesurements were first acquired on a SymbiaT6 gamma camera, then simulated with the same parameters on Simind. ACCT reconstruction was used to define the simulation geometries. Simulation geometries were Zubal-like phantoms (26, 27), built from the ACCT. ACCT data was segmented according to hounsfield units using MiM software (7.0, Cleveland). Images bit depth was set to 8 bits, and a unique value was attributed to each segmented areas using ImageJ sofware (28). Those values were used in Simind to set the desired value of density and radioactivity concentration in the defined areas. SPECT were simulated with the same parameters than the acquisitions. Photons emitted from ^99m^Tc decay were simulated with a 140 keV Energy and 88.5% abundance. As Krypton gas is continuously inspired and expired and has a very fast decay (half-life is 13 s), it was simulated as a stationary gas without significant decay, with homogeneous concentration, with a 190 keV energy and 100% abundance.

All SPECT reconstructions were performed on Siemens MI-Apps software, with FLASH3D, four iterations, eight subsets, 8.4 mm gaussian post-filtering, scatter correction (DEW), with and without attenuation correction.

In order to assess the simulated data, image quality tests were run on both simulated and aquired data, and compared in terms of Root Mean Square Deviatiation (RMSD) for spatial resolution and activity recovery (see Appendix 1).

### Lung V/Q SPECT Realistic Model

#### Simulation Parameters

Using the ACCT, five representative tissues were delineated using MiM Software (MiM 7.0, Cleveland), including outside air, bones, fat, soft tissues, lungs, and bronchi. A code was assigned to each area. Digital phantom was sub-sampled in a 128^2^ matrix to accelerate the simulation calculation, and the simulation grid was 128 × 128 × 108 matrix, corresponding to 3.92 × 3.92 × 3.59 mm voxels. With regards to ventilation, the simulated radioactivity was evenly set to 55 kBq·mL^−^in the lungs and the airways. With regards to perfusion, in order to model anterior to posterior gradient of the distribution of radioactivity on perfusion SPECT, lungs were divided into sixteen coronal planes. For each coronal plane a relative to maximum radioactivity concentration value was assigned. The values were set to fit the mean anterior to posterior intensity gradient measured on a normal cases database (29), from 43 kBq·mL^−1^ inside the first coronal plane to 65 kBq·mL^−1^ in the last coronal plane. As the source map is different when simulating ventilation and perfusion, simulations were not run simultaneously. Scatter data was stored at each energy window, and ^81m^Kr scatter was added to ^99m^Tc lower scatter and primary energy windows.

#### Reconstructions Analysis and Z-Score Maps

In order to assess the realism and consistency of simulated SPECT, reconstructed simulations were compared to a 73 co-registered normal cases database (29). Z-score maps were generated as follows: First, SPECT simulated reconstructions were normalized according to the mean value. Z-score map was then calculated at each voxel coordinate as follows:

$$\begin{array}{l} Z-score_{SPECT\;Reconstruction}\left(x,y,z\right)\\ \;=\frac{\left[Pixelvalue\left(x,y,z\right)-MEANmapValue(x,y,z)\right]}{SDmapValue(x,y,z)}; \end{array}$$

Z score histograms were computed in a ROI built 0.5 cm inside the lungs CT boundaries to avoid partial volume effect artifacts. Simulations were evaluated as the mean Zscore ± SD, and as the pourcentage of voxels below or above 1 SD.

#### Example of Application

As an example of the possible applications, a simulation with a segmental perfusion defect was performed. A segmental area was delineated on ACCT and the relative radioactivity concentration was set to 50% in this zone.

## Results

### Realistic Simulated Lung V/Q SPECT

**Figure 1:**
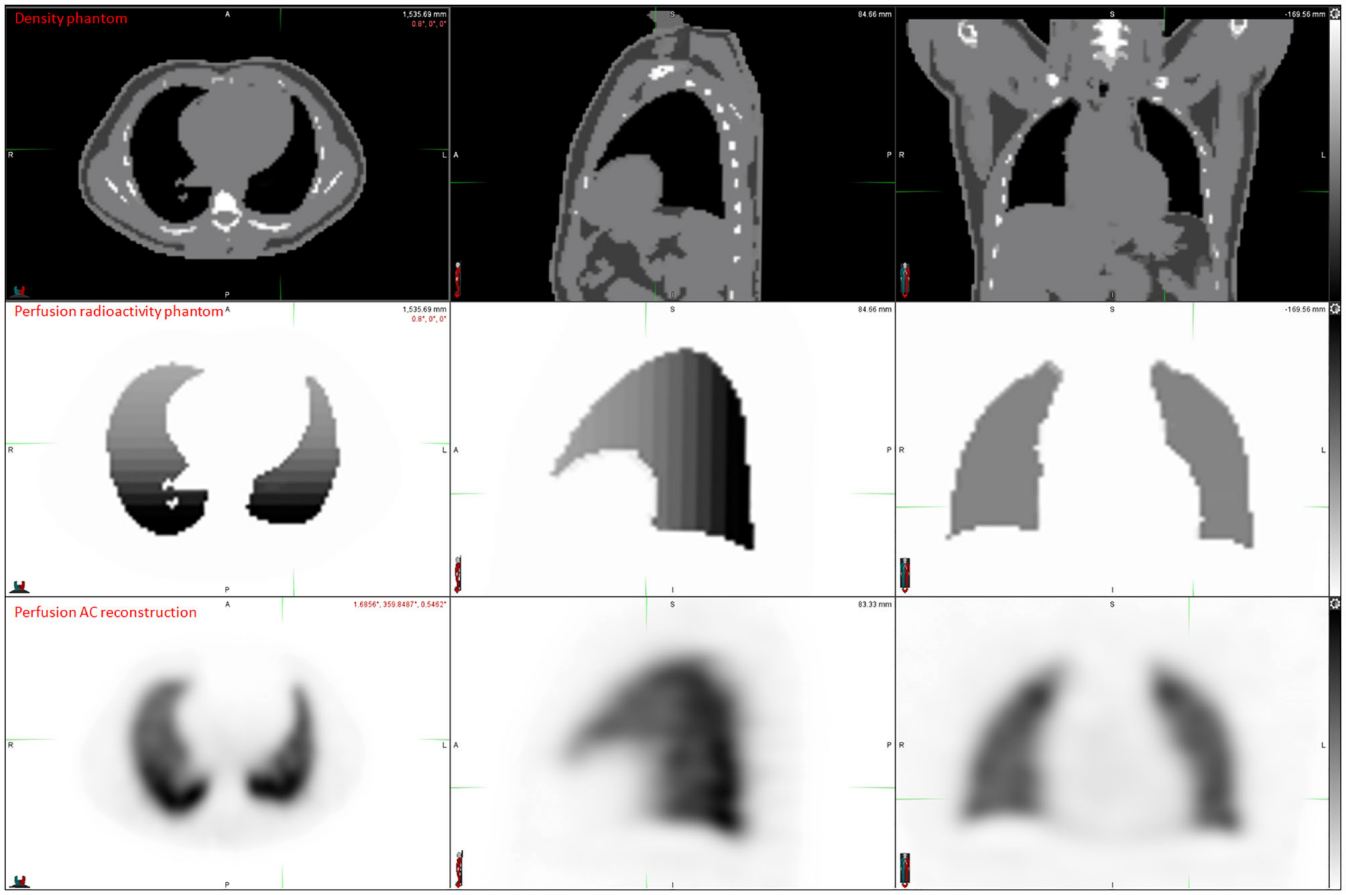
Digital phantom, radiactivity source map definition, AC and NoAC reconstructions of the simulated projections.

**Figure 2:**
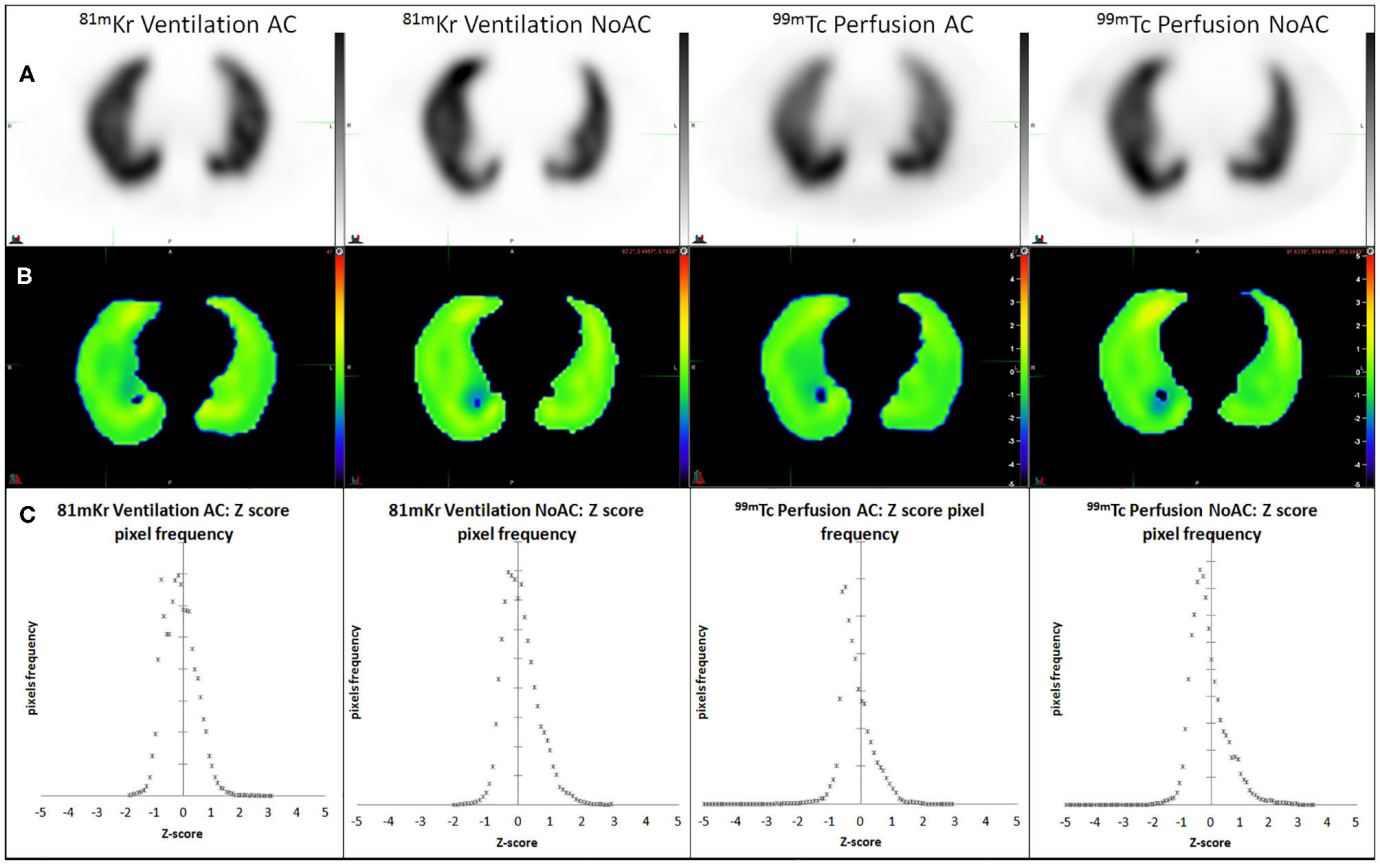
AC and NoAC reconstructions for ventilation and perfusion **(A)**, corresponding Z-score map **(B)** and pixels Z-score histograms **(C)**, and statistics measured 0.5 cm inside CT boundaries.

**Figure 3:**
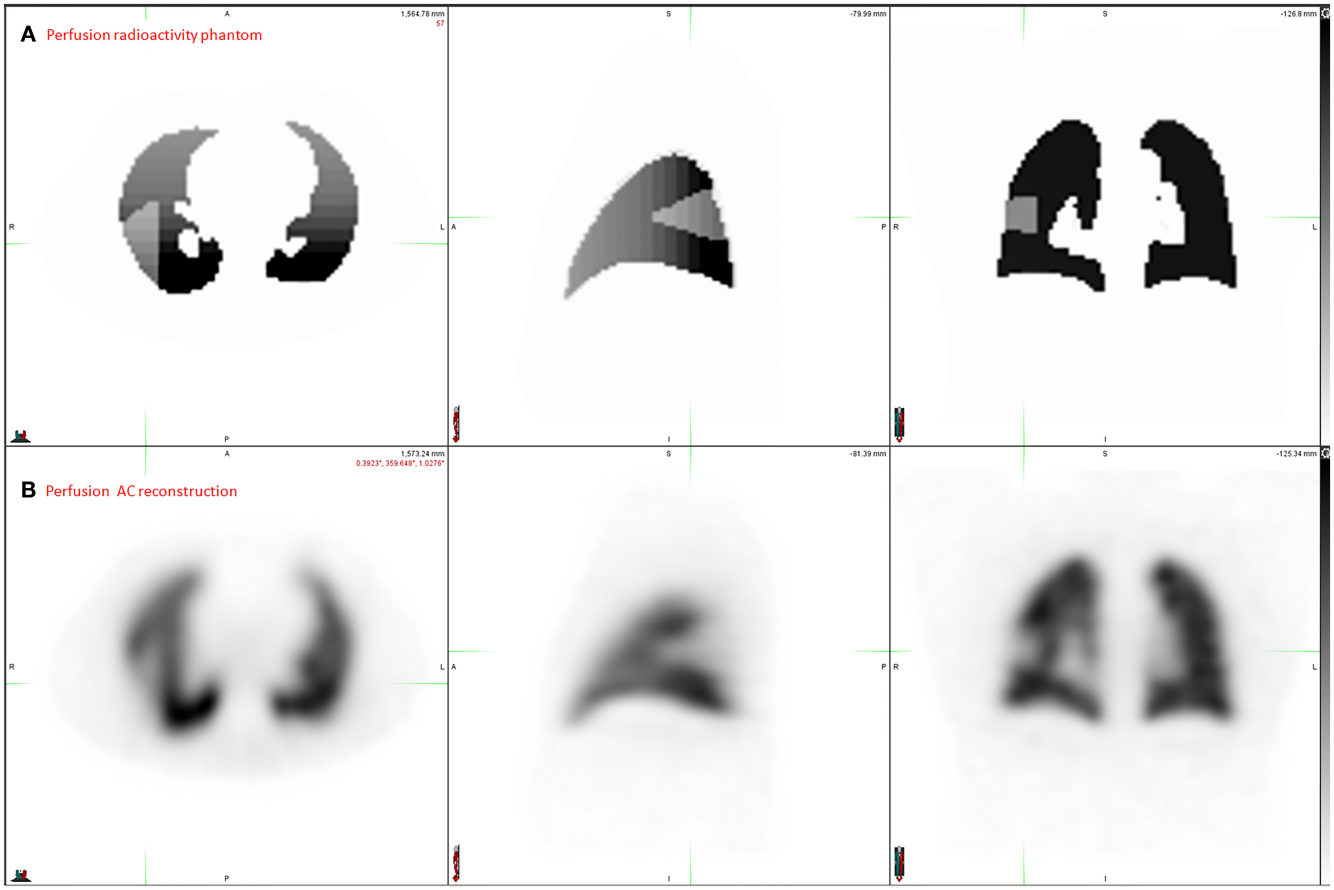
Application exemple: PE simulation. Radioactivity source map representing the ground Truth **(A)** and the corresponding perfusion AC reconstruction **(B)**.

Digital phantom, radioactivity source map and the corresponding AC and NoAC perfusion reconstructions are shown in Figure 1. An axial slice and the corresponding Zscore maps and 3D histogram for ventilation, perfusion, AC and NoAC reconstructions are shown in Figure 2. Zscores distribution for all reconstructions are summurized in Table 1. Mean Zscores ranged from −0.1 to −0.2. An exemple of PE simulation, its radioactive source map and the corresponding perfusion AC reconstruction is shown in Figure 3.

**Table 1:** Zscores distribution for ventilation AC, NoAC, and perfusion AC and NoAC.

	**Ventilation AC**	**Ventilation NoAC**	**Perfusion AC**	**Perfusion NoAC**
Mean Zscore (SD)	−0.2 (0.7)	−0.2 (0.7)	−0.2 (0.6)	−0.1 (0.6)
%pixels > 1	4.6	5.2	3.5	4.9
%pixels < −1	1.9	1.3	1.9	3.8

## Discussion

In this study we created a realistic model for dual isotopes V/Q SPECT-CT Monte-Carlo simulations. The gamma camera was correctly modeled, as shown by spatial resolution and contrast recovery measurements. Reconstructed simulations of ventilation and perfusion scans were very close to a normal cases database including 73 normal co-registered V/Q SPECT/CT (29).

The Monte-Carlo code used in this study was simind. As compared with Gate (30) or MCNPX (31) packages, this program is known to be fast, thanks to variance reduction techniques and analytic calculation of the collimator response. Taheri et al. showed that the accuracy was comparable to other packages (32). The use of a user friendly graphical user interface, CHANGE, and the ability to write scripts to run automatically simulations with different parameters makes Simind an easy package to run multiple SPECT simulations.

The system performances comparison showed slight differences between simulations and acquisitions. The root mean square deviation was lower than 1 mm for spatial resolution and lower than 1% for contrast recovery. Thus, the system was correctly modeled and usable to run realistic simulations, in accordance with published studies (33, 34). Energy resolution was not verifed, as it is an input of the simulation. Sensitivity was not verified either, as images are scaled before adding Poisson noise. This may have been of concern if the simulations were run in a dosimetric purpose, but not for producing images. In order to model the anterior to posterior intensity gradient, we defined 16 horizontal planes in the patient orientation, so that each plane's width was close to 1 cm (3 pixels). This is far under the spatial resolution for SPECT with a 30 cm radius, and thus is enough to model precisely the gradient. The radioactivity values in each plane was defined based on a parametric mean perfusion AC map obtained from the normal cases database (29). Based on Z-score analysis, V/Q SPECT modeling was satisfactory, with a mean Z value close to zero, and at least 91.3% of the pixels ranging from −1 to 1 SD. We measured Z scores in ROI defined 0.5 cm inside CT boundaries corresponding to a 1-pixel width in order to avoid false measurements due to partial volume effects on the edges of the Zscore map.

A current challenge in PE management is the ability to estimate the Pulmonary Vascular Obstruction Index (PVOI), which has been shown to be predictive of PE reccurence (3–5). In order to measure precisely the PVOI with V/Q SPECT, several methods have been proposed (11–13, 35), but an accurate evaluation remains limited by the the lack of ground truth. We illustrated the interest of a realistic model of lung V/Q SPECT-CT Monte-Carlo simulations in Figure 3. A knowledge of the real volume and geometry of perfusion defects, for various morphologies and anterior to posterior gradient intensities, should allow to develop and test new delineation methods, such as statistical map threshold.

Our study has some limitations. First, we used a static Zubal-like phantom instead of dynamic X-CAT phantom. Although respiratory motion is a challenge in PET imaging, it has less impact on SPECT images because of the lower spatial resolution of the technique. Indeed, based on the analysis of 73 normal co-registrated cases database (29), the impact of respiratory motion on the uptake variability in the basis areas was very low, especially as compared with the anterior to posterior gradient. Second, the DEW scatter correction method is not the more efficient, since both scatter (from ^99m^Tc itself) and downscatter (from ^81m^Kr) are present in the main Tc-window around 140 keV. It has been shown that triple energy windows method (TEW) is better in the case of multiple energy isotopes (36), or here in multiple isotopes SPECT. However, given that the normal cases database was created with the DEW method, the reconstructions of the simulations were performed with the same method to avoid a bias in Z-score analysis. Third, we did not model the mild gradient described by some authors in the caudo-cranial direction (35). Similarly, this gradient was negligible in the 73 normal co-registrated cases database (29). Fourth, some more singularities have been described, such as the fissure sign or the segmental contour sign (37). The distribution of ^99m^Tc-MAA often does not extend to the periphery of a segment or lobe, often finishing 1 cm or less before the anatomical boundary. This is usually attributed to the lack of pulmonary artery supplied perfusion to the peripheral surface of the lung which is supplied by the bronchial circulation. This was not simulated in our model. Finally, Walrand et al. showed that it was possible to simulate regionnal heterogeneity of liver perfusion with ^90^Y-microspheres, taking into account the diameter of arteries (38). Similarly, simulating the physics of MAA particles inside the pulmonary arterial tree may have improved the realism of simulations. However, these simulations were not developed for dosimetry purposes.

## Conclusion

We developed a realistic model for dual isotopes lung V/Q SPECT-CT, integrating the anterior posterior gradient on perfusion images. This model can be used to build a catalog of clinical scenarios, in order to test delineation methods of functionnal lung volumes. In the context of PE, this could help to develop new delineation methods for PVOI estimation.

## Data Availability Statement

The raw data supporting the conclusions of this article will be made available by the authors, without undue reservation.

## Author Contributions

DB, PL, and PS contributed to designing the study. DB, LW, ME, PR, RL, PL, and PS contributed to managing the imaging procedures. DB, LW, ME, PL, and PS contributed to analyzing the data. All authors contributed to writing the manuscript, read, and approved the final manuscript.

## Conflict of Interest

The authors declare that the research was conducted in the absence of any commercial or financial relationships that could be construed as a potential conflict of interest.
