# Supplementary material for: Normal Dual Isotope V/Q SPECT Model for Monte-Carlo Studies
Source: Front Med (Lausanne). 2020 Aug 18;7:461. doi: 10.3389/fmed.2020.00461 (PMC7461964; doi:10.3389/fmed.2020.00461)
Supplement: Supplementary file 1 [file Data_Sheet_1.PDF]

Appendix 1 : Simulated image quality assessement

Methods

In order to assess the simulated data, image quality tests were run on both simulated and aquired data, and compared [25, 33, 38]

Spatial resolution

Spatial resolution was measured with static images of a ^{99m}Tc point source, with a 5, 10, 20 and 30cm source to detector distance, with 1.2mm pixels. Source activity was 26 MBq at acquisition time. An activity profil was measured throw the center of the point image and was fitted with a gaussian function to calculate spatial resolution. Root mean square Deviation (RMSD) was calculated between acquired and simulated spatial resolutions (SR) as follow:

$$\boldsymbol{RMSD} = \sqrt{\frac{\sum_{i=1}^{4}\left(\left(SR_{simulation_i}\right)-\left(SR_{acquistion_i}\right)\right)^2}{4}}$$

Contrast recovery

SPECT acquisition and simulation were performed on a NEMA IEC. The Phantom was filled with a ^{99m}Tc solution. Activity concentration was 480 kBq.mL^{-1} in the hot spheres and 45 kBq.mL^{-1} in the background compartiment. Spheres, background compartiment and plastic were segmented to generate the digital phantom and hot spheres and background were simulated. On SPECT reconstructions, regions of interest (ROI) around the spheres were defined using the CT data and multiple background ROIs were defined in the low area of the phantom within the slice containing the center of the spheres. Contrast recovery was calculated for all spheres i as follow: CR_i=(sphere counts mean value / background counts mean value) / (sphere to background activity ratio), and Root Mean Square Deviation (RMSD) was calculated.

Results

RMSD between acquired and simulated spatial resolutions at 5, 10, 15 and 20 cm was 0.7 mm. RMSD of the contrast recovery measured in the six hot spheres was 0.8%.

Tab. 2: Gamma camera performances verification

		Acquired	Simulated	RMSD
**Spatial resolution**	5 cm	9.0 mm	8.1 mm	0.7 mm
	10 cm	11.8 mm	11.3 mm	
	20 cm	18.5 mm	17.8 mm	
	30 cm	25.1 mm	25.0 mm	
**Contrast Recovery**	26.5 mL	66.0 %	64.8 %	0.8 %
	11.5 mL	52.5 %	52.7 %	
	5.6 mL	31.1 %	29.9 %	
	2.6 mL	19.0 %	20.0 %	
	1.2 mL	13.5 %	13.1 %	
	0.5 mL	10.5 %	11.3 %	
